# Practitioner perceptions on the use of exercise and nutritional interventions for patients with breast cancer receiving radiation therapy

**DOI:** 10.1002/jmrs.713

**Published:** 2023-08-10

**Authors:** Laura Feighan, Lesley MacDonald‐Wicks, Robin Callister, Yolanda Surjan

**Affiliations:** ^1^ Global Centre for Research and Training in Radiation Oncology, School of Health Sciences, College of Health, Medicine, and Wellbeing The University of Newcastle Callaghan New South Wales Australia; ^2^ School of Health Sciences, College of Health, Medicine, and Wellbeing The University of Newcastle Callaghan New South Wales Australia; ^3^ School of Biomedical Sciences and Pharmacy, College of Health, Medicine, and Wellbeing The University of Newcastle Callaghan New South Wales Australia

**Keywords:** breast, cancer, patient care, radiation effects, radiation oncologist, radiation therapist, radiotherapy (radiation therapy)

## Abstract

**Introduction:**

Radiation therapy treatment for breast cancer may negatively impact patients' health‐related quality of life. Evidence suggests exercise and nutrition interventions may be beneficial to patients experiencing compromised health‐related quality of life. This study investigates whether radiation oncology practitioners support the implementation of a tailored exercise and nutrition intervention for patients and explores their interest in participating in training for exercise and nutrition as interventions.

**Methods:**

Data were collected by an online survey, deployed to public and private radiation oncology departments, across three Australian states (Australian Capital Territory, New South Wales, Queensland). The survey was completed between June and August 2020. Radiation oncologists, radiation oncology registrars, radiation therapists and radiation oncology nurses completed the survey. The survey included demographics, patient assessment and questions regarding the radiation oncology practitioners' use of exercise and nutrition as interventions.

**Results:**

Of 192 practitioners targeted, 76 completed the survey, for a response rate of 40%. Of 76 respondents, 42% ‘sometimes’ recommended exercise and 41% ‘sometimes’ recommended nutrition as health‐related quality of life interventions to their patients. The majority indicated they would benefit from more training in these subjects, with 58% for exercise and 55% for nutrition. 47 per cent of respondents thought patients would benefit from a tailored exercise and nutrition programme and 62% agreed they would refer patients to a programme if it were available.

**Conclusions:**

Radiation oncology practitioners reported they need training in exercise and nutrition to better understand how this can benefit the health‐related quality of life of breast cancer patients. Also, the findings indicate that if such an exercise and nutrition intervention were readily available, practitioners would refer patients who may benefit from this intervention.

## Graphical abstract

This study investigates if radiation oncology practitioners support the implementation of a tailored exercise and nutrition intervention for patients with breast cancer receiving radiation therapy. It also explores their interest in participating in training for exercise and nutrition as interventions.

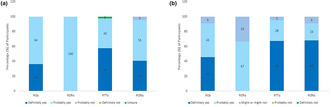

## Introduction

Over time, due to medical and technological advances, the breast cancer survival rate has improved significantly. In Australia between 2013 and 2017, it was estimated that the chance of surviving breast cancer for 5 years was 92%.^1^ A patient with breast cancer experiences a long treatment journey, generally beginning with surgery (lumpectomy or mastectomy), followed by adjuvant chemotherapy and/or radiation therapy (RT).^2^ The side effects of RT for breast cancer can include RT‐induced skin reactions, fatigue, changes in breast skin colour and thickness, swelling and tenderness of the breast, insomnia and loss of appetite.^3^ Alongside side effects, RT can cause the patient to experience emotional and psychosocial issues.^4^

Flowers et al. found body image concerns are reported by up to two thirds of breast cancer survivors.^5^ Przezdziecki et al. explain body image as a person's perception of their physical appearance that contributes to their self‐worth. When body image is negatively altered it can compromise both physical and emotional health.^6^ The loss of a breast (or both when necessary), breast alignment changes, disfigurement and scarring all contribute to this concern.^3^ Furthermore, breasts are fundamentally connected to a woman's individuality and sexuality, which can be heavily impacted by breast cancer treatment.^6^

Kim et al. and Stiegelis et al. report anxiety and depression as the most common psychological complications experienced by patients receiving RT treatment. The symptoms can include depressive moods, difficulty concentrating, trouble sleeping, behavioural changes and self‐isolation.^7^, ^8^, ^9^ Furthermore, there is a distinct relationship between depression and cancer‐related fatigue (CRF). Berger et al. report CRF being experienced by up to 80% of patients receiving RT treatment. The impact of CRF on regular daily activities can affect the capacity to work, maintain financial security, enjoy leisure activities and the patient's quality of life (QoL).^10^

Nutritional depletion in cancer patients has been associated with psychosocial factors. Equally, psychological complications such as anxiety and depression can cause loss of appetite and decrease nutritional intake.^11^ Canetti et al. discussed the relationships between diet and emotions, in particular the link between the tendency to eat healthy food when feeling positive and eating food low in nutritional value but high in energy, saturated fat, sugar and salt when feeling sad. Similarly, the relationship between the quantity and frequency of meals related to emotion is considered; during times of negative emotion, impulsive eating may occur.^12^

The amalgamation of complications caused by RT treatment can ultimately affect a patient's quality of life (QoL). More specifically, the physical, psychological and social facets of health are known as health‐related quality of life (HRQoL).^13^ With increasing breast cancer survival, a greater focus on the HRQoL consequences of RT treatment is warranted.

There is evidence that exercise and nutrition (E&N) interventions benefit HRQoL.^14^, ^15^ Biddle et al. stated anxiety, stress, depression, self‐esteem, mood, cognitive function and psychological adjustment have been shown to have at least a moderate level of benefit from exercise.^16^ Studies by Mustian et al. and Campbell et al. assessed the benefit of supervised aerobic exercise on patients with breast cancer to improve HRQoL during RT treatment. These studies demonstrated significant improvement from baseline to post‐intervention in CRF, anxiety, depression and physical functioning.^17^, ^18^ In addition, the use of dance movement, qigong exercise and yoga have been shown to be beneficial in the intervention of stress, anxiety, sleep quality and mood states in patients with breast cancer receiving RT.^19^, ^20^, ^21^

Nutritional interventions are also beneficial. Breast Cancer Network Australia et al. suggest patients with breast cancer maintain a healthy, balanced diet during treatment. This is defined as a diet high in vegetables, fruits, legumes with whole grains, bread, rice, pasta, lean meats and reduced fat dairy. Also, limiting intake of saturated fat, salt, foods high in sugar and alcohol.^3^ Despite existing evidence of E&N benefitting patients, it is unclear how knowledgeable radiation oncology practitioners are regarding E&N interventions for patients receiving RT treatment. The aim of this study was to identify radiation oncology practitioners' use of E&N for managing RT side effects of patients with breast cancer, including considerations of HRQoL, perceptions on referring patients to E&N intervention programmes and partaking in E&N training.

## Methods

### Settings and participants

A survey was designed to target radiation oncology practitioners who have regular patient interaction. The participants included radiation oncologists, radiation oncology registrars, radiation therapists and radiation oncology nurses. An invitation was sent to 13 radiation oncology department site managers, requesting they share survey information, including a Participation Information Statement, with their staff. A link to an online survey platform was provided. The survey was shared between June and August 2020 to 192 practitioners across three Australian states (Australian Capital Territory (ACT), New South Wales (NSW), Queensland (QLD)). Ethical approval for the study was granted by the Hunter New England Human Research Ethics Committee (2019/ETH00345).

### Survey

**Figure 1:**
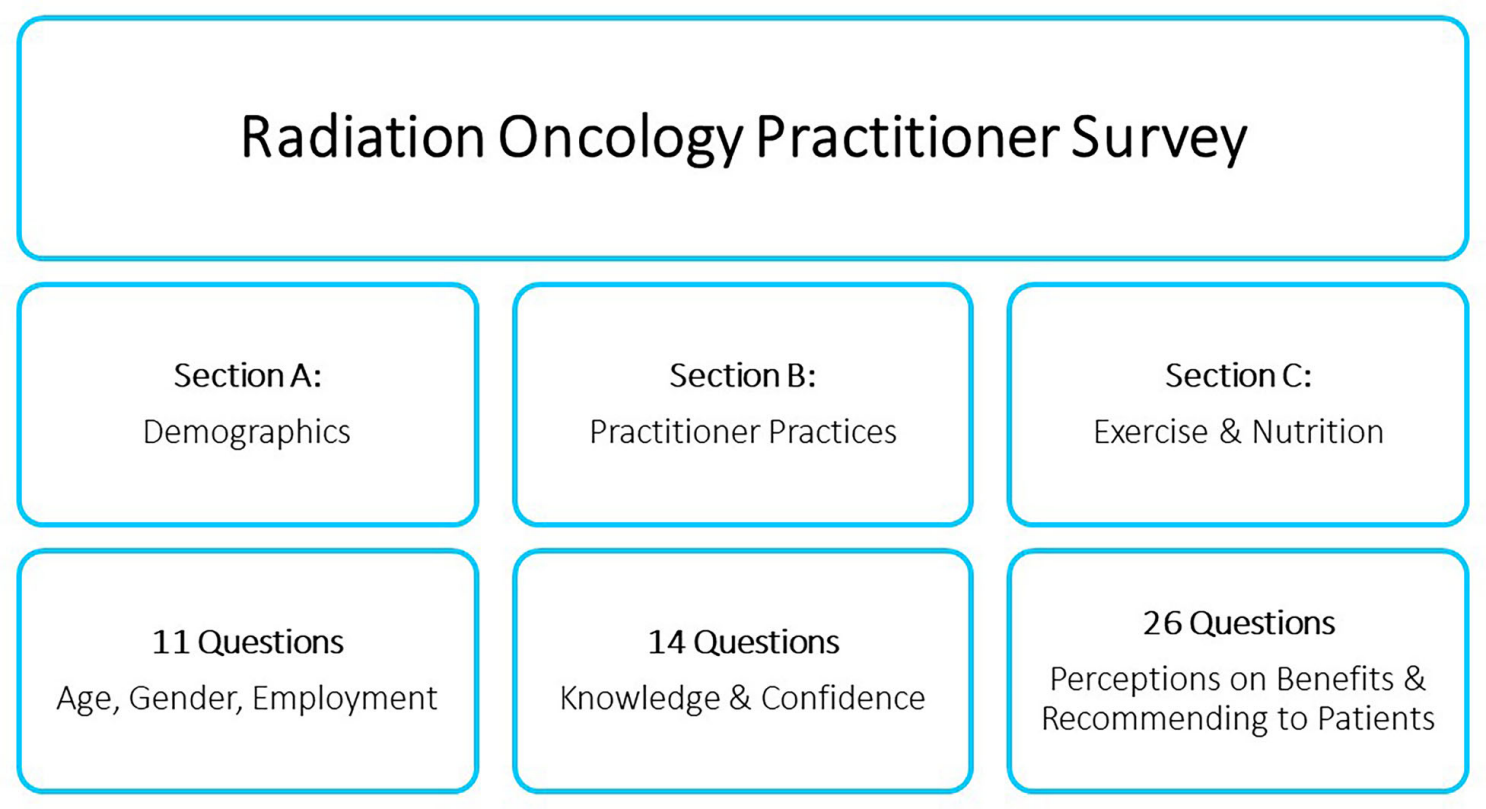
Summary of survey sections completed by practitioners in ACT, NSW and QLD in 2020.

The survey was delivered through Qualtrics, an online platform ‘(Qualtrics XM//The Leading Experience Management Software, Level 15, 1 Denison Street, North Sydney, NSW, 2060, Australia). It consisted of 51 multiple choice questions (see Supporting Information, Data S1). Participants were informed the survey would take approximately 10 min to complete. Figure 1 displays a summary of the survey, including the sections and question totals. For the purpose of this paper, questions regarding the practitioners recommending E&N interventions to their patients are reported, alongside their perceptions on patients benefitting from a tailored E&N programme and perceptions on participating in training for E&N as interventions for both side effects and HRQoL.

### Data analysis

Data from the responses were exported from Qualtrics to Microsoft Excel for analysis. Upon export, responses from each radiation oncology discipline were isolated to display comparison. As questions were asked for exercise and nutrition separately, these responses were merged side by side in Microsoft Excel. Data analysis consisted of counts (also expressed as frequencies) to provide descriptions of the responses. Data were presented as stacked bar charts.

Kruskal–Wallis tests were performed to determine whether there were statistically significant differences amongst the practitioners' responses to the survey questions. The significance level used was *P* < 0.5.

## Results

### Participants

Of the 192 practitioners targeted, 76 completed the survey for a response rate of 40%. Table 1 provides demographic information for the 76 participants. The main professional disciplines were radiation therapists (53% (*n* = 40)) and radiation oncology nurses (29% (*n* = 22)). Most participants (83% (*n* = 63)) were between 25 and 54 years of age, female (75% (*n* = 57)) and worked full time (67% (*n* = 51)). Most participants (61% (*n* = 46)) had less than 10 years practice in their profession.

**Table 1:** Demographic characteristics of the respondents. Data are presented as counts and percentage of the 76 respondents.

Characteristics	Responses N (%)
Professional discipline	
Radiation Oncologists	11 (14%)
Radiation Oncology Registrars	3 (4%)
Radiation Therapists	40 (53%)
Radiation Oncology Nurses	22 (29%)
Age (Years)	
20–24	6 (8%)
25–34	30 (40%)
35–44	17 (22%)
45–54	16 (21%)
>55	7 (9%)
Gender	
Male	19 (25%)
Female	57 (75%)
Years of Experience	
0–5	28 (27%)
6–10	18 (24%)
11–15	12 (16%)
16–20	8 (11%)
21–30	5 (6%)
>30	5 (6%)
Employment	
Casual	5 (7%)
Part‐time	20 (26%)
Full time	51 (67%)

### Statistical analysis results

The statistical analysis of practitioner responses to each survey question reported found no statistically significant differences among the practitioner groups. The radiation oncology registrars' data were not included in these analyses due to the very small number of participants. This lack of significant findings may be in part due to the relatively small number of survey participants in each practitioner group. The response patterns described below are qualitative trends, not statistically significant findings.

### Recommending exercise and nutrition as interventions

**Figure 2:**
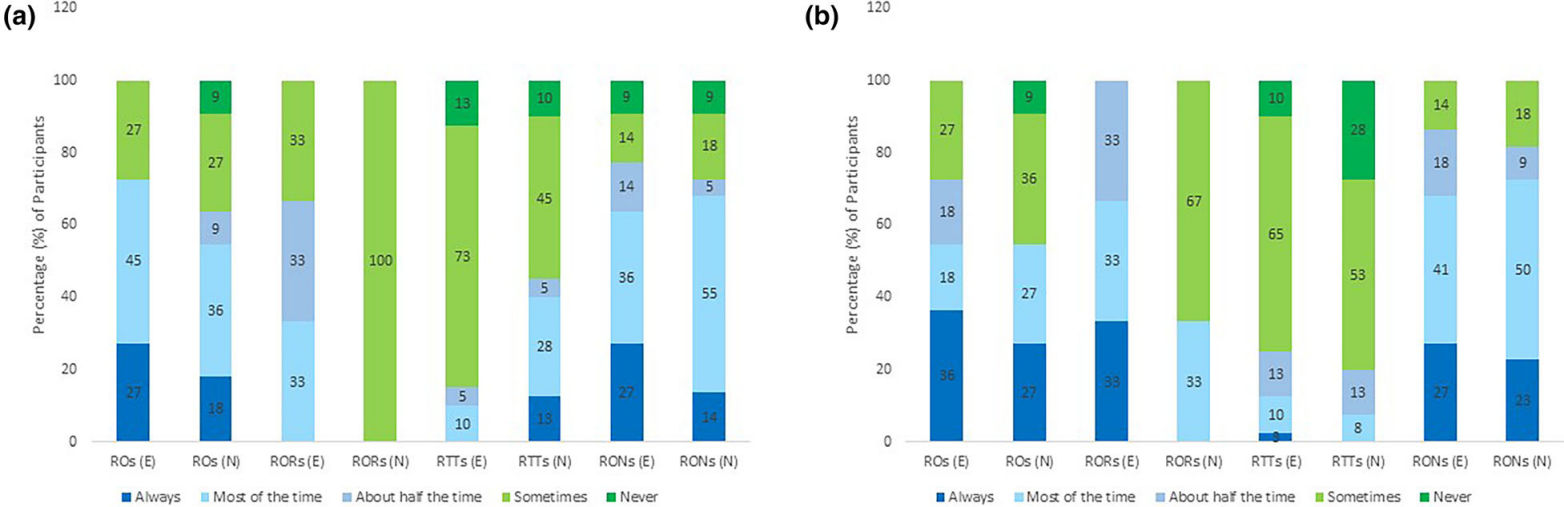
(A) Proportion of response options provided by each practitioner group regarding recommending exercise and nutrition as interventions to their patients with breast cancer for radiation side effects. (B) Proportion of response options provided by each practitioner group regarding recommending exercise and nutrition as interventions to their patients with breast cancer for compromised health‐related quality of life due to radiation therapy.

Figure 2A,B indicate the response profiles of practitioners regarding their recommendations of exercise and nutrition interventions for side effects or health‐related quality of life. For example, in Figure 2A among radiation oncologists, 27% (*n* = 3) ‘always’ recommend exercise for side effects, 45% (*n* = 5) recommend exercise for side effects ‘most of the time’ and 27% (*n* = 3) ‘sometimes’ recommend exercise for side effects.

Collectively, the most frequent response was ‘sometimes’ for recommending interventions for RT‐related side effects and for compromised HRQoL. The recommendation of exercise and nutrition for RT‐related side effects (‘sometimes response’) ranged between 37 and 47%, respectively, and between 41 and 42% for HRQoL, respectively.

As shown in Figure 2A,B, radiation oncologists reported they recommend (‘most of the time’) exercise and nutrition for RT side effects (between 36 and 45%, respectively) and ‘always’ recommend exercise and nutrition for comprised HRQoL (between 27 and 36%, respectively). Of the oncologists, 9% (*n* = 1) ‘never’ recommend nutrition as intervention for either side effects or HRQoL.

The registrar's most frequent response for nutrition intervention was ‘sometimes’ for both side effects (100% (*n* = 3)) and HRQoL (67% (*n* = 2)), and they recommend exercise as an intervention for HRQoL more than side effects, with 33% (*n* = 1) ‘always’ recommending exercise.

Radiation therapists are less likely to recommend E&N as interventions. The majority ‘sometimes’ recommend E&N as interventions for both side effects and HRQoL. The recommendation of E&N for RT‐related side effects (‘sometimes response’) ranged between 45 and 73%, respectively, and for HRQoL ranged between 53 and 65%, respectively. Interestingly, 13% (*n* = 5) responded ‘always’ to recommending nutrition as a side effect intervention and 3% (*n* = 1) for exercise as a HRQoL intervention (Fig. 2A,B), however there was no response for ‘always’ recommending exercise as a side effect intervention or nutrition for HRQoL.

For nurses, the most frequent response for recommending E&N as interventions for both side effects and HRQoL was ‘most of the time’, the recommendation for side effects ranged between 36 and 55%, respectively, and HRQoL ranged between 41 and 50%, respectively.

Several radiation therapists and nurses responded ‘never’ to both E&N as interventions for RT side effects – with 13% (*n* = 5) of radiation therapists never recommending exercise, 10% (*n* = 4) never recommending nutrition and 9% (*n* = 2) of nurses never recommending either exercise or nutrition.

### A tailored exercise and nutrition programme as a standard intervention

**Figure 3:**
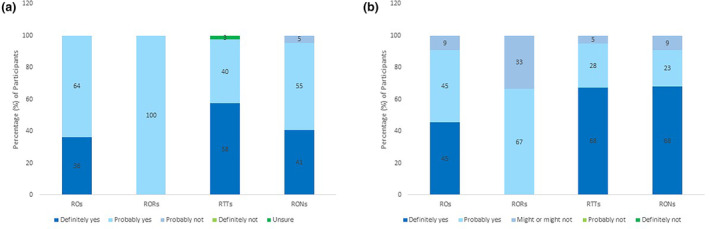
(A) Practitioners perceptions on their patients benefitting from a tailored exercise and nutrition programme. (B) Practitioner perceptions on referring patients to a tailored exercise and nutrition programme.

Figure 3A indicates response profiles related to whether practitioners thought patients would benefit from a tailored exercise and nutrition programme. For example, among radiation oncologists 36% (*n* = 4) responded ‘definitely yes’, and the other 64% (*n* = 7) responded ‘probably yes’. Figure 3B indicates response profiles related to whether practitioners would refer patients to a tailored exercise and nutrition programme. For example, among radiation therapists 68% (*n* = 27) responded ‘definitely yes’, 28% (*n* = 11) responded ‘probably yes’ and 5% (*n* = 2) responded ‘might or might not’.

The majority of radiation oncologists (64% (*n* = 7)), registrars (100% (3)) and nurses (55% (*n* = 12)) responded ‘probably yes’ and the majority of radiation therapists (58% (*n* = 23)) responded ‘definitely yes’. Only 5% (*n* = 1) of nurses responded, ‘probably not’ and 3% (*n* = 1) of radiation therapists were ‘unsure’ (Fig. 3A). Many radiation oncologists (45% (*n* = 5)), therapists (68% (*n* = 27)) and nurses (68% (*n* = 15)) responded ‘definitely yes’ and the majority of registrars (67% (*n* = 2)) responded ‘probably yes’ to referring patients to said programme if it were available. All disciplines had responses of ‘might or might not’ refer patients to a tailored E&N programme (Fig. 3B).

### Education for practitioners in exercise and nutrition

**Figure 4:**
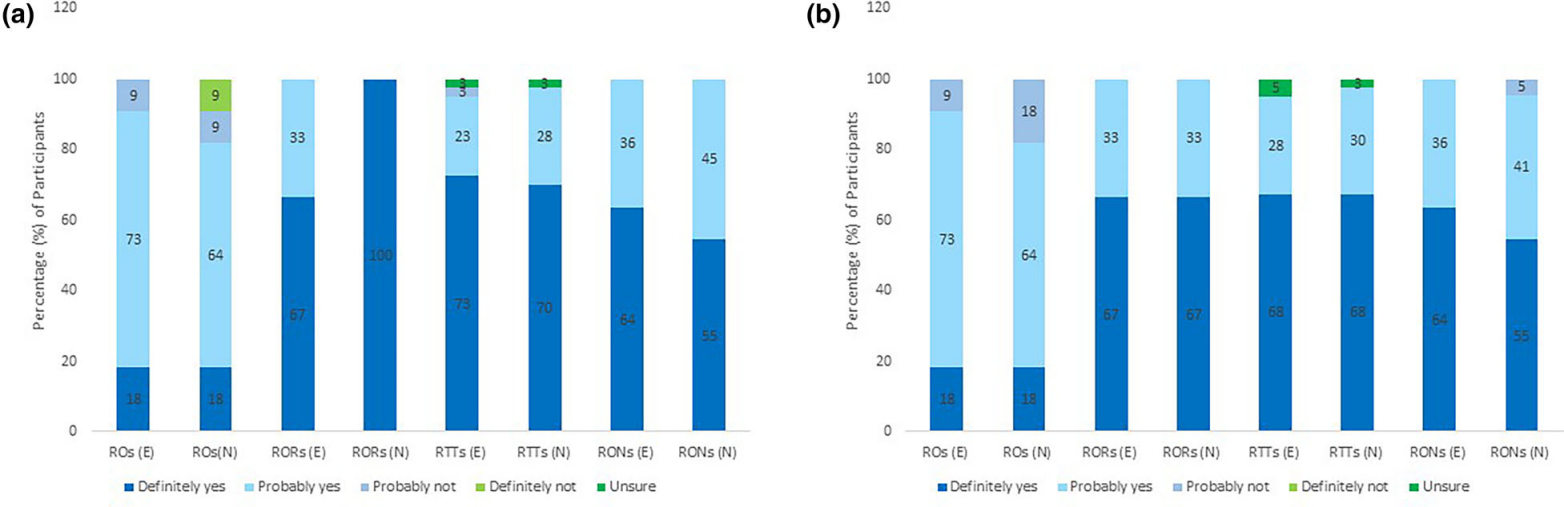
(A) Practitioner perceptions on participating in training regarding exercise and nutrition as interventions for patients with radiation therapy side effects. (B) Practitioner perceptions on participating in training regarding exercise and nutrition as interventions for patients with compromised health‐related quality of life, due to radiation therapy.

Figure 4A,B indicate response profiles for practitioner's perceptions of participating in training regarding exercise and nutrition interventions for addressing side effects or health‐related quality of life. For example, in Figure 4A among radiation oncology registrars, 67% (*n* = 2) responded ‘definitely yes’ to participating in training for exercise as side effect intervention, while the remaining 33% (*n* = 1) responded ‘probably yes’. In Figure 4B among radiation oncology nurses, 55% (*n* = 12) responded ‘definitely yes’ to participating in training regarding nutrition as health‐related quality of life intervention, 41% (*n* = 9) responded ‘probably yes’ and remaining 5% (*n* = 1) responded ‘probably not’.

Collectively, practitioners' responses to participating in training in E&N as interventions were similar for both side effects and HRQoL. Most practitioners responded ‘definitely yes’ with 62% (*n* = 47) for exercise and 59% (*n* = 45) for nutrition as side effect interventions and 59% (*n* = 45) for exercise and 57% (*n* = 43) for nutrition as HRQoL interventions.

Radiation oncologists' responses were effectively the same for side effects and HRQoL, with the majority responding ‘probably yes’ for exercise (73% (*n* = 8)) and nutrition (64% (*n* = 7); Fig. 4A,B). Of the radiation oncologists, 9% (*n* = 1) responded ‘definitely not’ to taking part in training in nutrition as intervention for RT‐related side effects, being the only profession to use this answer overall. With responses of ‘probably not’ and ‘definitely not’, oncologists were least interested in participating in training.

The registrars' responses demonstrated they are the most interested in receiving training, with between 67 and 100% responding ‘definitely yes’ to participating in training for E&N, respectively, as an intervention for side effects. For HRQoL, most registrars (67% (*n* = 2)) responded ‘definitely yes’ for both E&N.

Radiation therapists responded ‘definitely yes’ to participating in training for E&N as side effect interventions (between 70 and 73%, respectively) and as HRQoL interventions (68% (*n* = 27, respectively)). The radiation therapists were the only profession to respond ‘unsure’ to participating in training for both E&N interventions for side effects and HRQoL. Nurses' responses were evenly distributed between ‘definitely yes’ and ‘probably yes’ for E&N as interventions for both side effects and HRQoL, however 5% (*n* = 1) responded ‘probably not’ for nutrition as intervention for HRQoL. Collectively, over 55% of the practitioners would participate in training for E&N as interventions for radiation therapy side effects and compromised HRQoL.

For further analysis, all practitioner responses are provided as table presentations, attached as Tables S1–S3.

## Discussion

This study investigated whether radiation oncology practitioners recommend exercise and nutrition as interventions for the side effects and compromised HRQoL of patients with breast cancer receiving RT. Overall, the responses indicated that current practices regarding recommending E&N interventions varied between disciplines. In contrast, there was substantial consistency in the perceived necessity and desire for education in these subjects and for a standard approach to these interventions being implemented.

### Promotion of exercise and nutrition

Based on the results from this study, it is evident that the promotion of E&N is not consistent, with diverse responses amongst disciplines. While radiation oncologists ‘most of the time’ or ‘always’ recommend E&N, some radiation therapists and nurses ‘never’ recommend either. In a focus group study by James‐Martin et al. oncology professionals and patients were interviewed regarding diet, exercise and weight management. Of the themes investigated, the ‘information’ theme reported that practitioners were only offering information on these topics when it was requested by patients. Another theme, ‘patient and practitioner recommendations’ found that patients would like more specific information regarding diet, exercise and weight management.^22^ A similar request was cited in a review by Mustian et al. where patients reported they would prefer their oncology practitioners instigate discussion about exercise with them while receiving their cancer treatment.^23^ Furthermore, a study by Cantwell et al. explored healthcare professionals' promotion of physical activity in cancer care. The study (*n* = 43) included oncologists, nurses and allied health oncology specialists. They reported that 74% (*n* = 32) recommended physical activity to more than 75% of their patients with breast cancer.^24^

### Time and workforce

Cameron et al. explored the implementation of senior radiographers as advanced practitioners, by which they took responsibility for patients with breast cancer's ‘review clinics’, providing an opportunity to advance their skills, boost self‐confidence and enhance job satisfaction. It was discussed that undertaking review clinics was different to regular interactions the radiographers had with patients, as there is normally limited time for the patient to reveal personal problems during treatment. It was further discussed that guidance on diet and exercise (alongside other side effect interventions) would be useful to discuss with patients during these clinics.^25^ In addition, a study by Nadler et al. reported responses to a survey completed by 120 oncology care providers where 43% (*n* = 51) reported the lack of time practitioners have during their regular sessions with patients is a significant barrier to promoting exercise appropriately.^26^ This was also an obstacle reported by Hardcastle et al. in an international survey, where 34% (*n* = 38) of oncologists reported lack of time as a reason for not promoting physical activity.^27^ A study by Schmitz et al. discusses the disparity between the evidence supporting the necessity for exercise referral from oncology practitioners compared with the workforce required to initiate these interventions, suggesting that expansion of the oncology workforce is required to fully understand the benefit of exercise during cancer treatment.^28^ In our study, responses indicated that radiation oncology practitioners' recommendations around E&N as interventions were varied. In fact, a small number of oncologists, therapists and nurses responded ‘never’ to recommending E&N for side effects (9% (*n* = 7), respectively), exercise for HRQoL (5% (*n* = 4)) and nutrition for HRQoL (16% (*n* = 12)). These results could associate with the studies by Nadler et al. and Hardcastle et al. suggesting a reason for practitioners not recommending E&N to their patients is due to the lack of time during their regular scheduled sessions.

### Availability of resources

The exercise behaviour of patients with breast cancer, as a result of oncologists' recommendations, was investigated by Jones et al. Patients were randomly assigned to either an oncologist exercise recommendation only, oncologist exercise recommendation with a referral, or usual care. Results indicated that oncologists' recommendation of exercise, and more specifically, oncologists referring patients to exercise specialists, is a beneficial approach to encouraging engagement with exercise by patients with breast cancer during adjuvant treatment.^29^ Cantwell et al. reported close to 88% (*n* = 38) of oncology specialists agree their role includes encouraging their patients to engage in physical activity, despite the many barriers involved, including limited community‐based exercise programmes available for patient referral.^24^ Similarly, Nadler et al. reported 51% (*n* = 61) of oncology care providers *‘did not have the knowledge on how or where to refer a patient to exercise’*.^26^ In the ‘information’ theme of James‐Martin et al. study, practitioners reported that one of the key reasons for not providing information to patients included having insufficient resources on exercise, diet and weight management. Results from our study report that the majority of radiation oncology practitioners collectively responded with ‘definitely yes’ (61.8% (*n* = 47)) to referring patients to a tailored E&N programme as a standard intervention (if it were available). These results suggest that practitioners would like to refer patients to said programmes, despite the limited availability. Interestingly, results from our study also display a disparity in the recommendation of exercise versus nutrition regarding side effect intervention, with 47% of practitioners (‘sometimes’) recommending exercise for side effects compared to 37% (‘sometimes’) recommending nutrition. This is consistent with a study by Ligibel et al. where 971 oncology practitioners were surveyed regarding their evaluation of patients' body weight, physical activity and diet behaviours including referrals to related programmes. It was found that while practitioners were reviewing patients body mass index (BMI) and physical activity level regularly, they were less inclined to review a patient's diet.^30^ Likewise, Steele et al. discussed patient–practitioner communication in regard to diet, physical activity and BMI in both the general population and cancer patients. They reported less than 35% of cancer patients received diet improvement advice and less than 50% of patients received practitioner recommendations for physical activity.^31^

### Practitioner education

Dempsey et al. explored the willingness of radiation therapists to participate in training for nutritional support of patients receiving treatment to the gastrointestinal tract and genitourinary system, to which 100% (*n* = 21) were interested and 62% (*n* = 13) indicated that it would also increase their job satisfaction.^32^ Likewise, a qualitative study by Avancini et al. interviewed 14 nurses in focus groups to identify their perception of physical activity promotion in cancer patients, reporting one of the main barriers being insufficient training regarding the subject of physical activity.^33^ This barrier was also discussed by Nadler et al. where 33% (*n* = 40) of the oncology care providers ‘*did not feel qualified to discuss exercise or refer patients to a program’* and in the survey by Hardcastle et al. where oncologists reported a ‘*lack of training or guidance on exercise prescription’*.^26^, ^27^ This is consistent with the results of our study, as the majority of practitioners responded ‘definitely yes’ to participating in training, suggesting there is a necessity for training and education in the subjects of E&N for radiation oncology practitioners.

### Limitations

A limitation of this study includes the survey being distributed in ACT, NSW and QLD only; however, this was a preliminary dissemination, and it is intended that the survey will be undertaken by practitioners in the remaining states in the future. In addition, this survey design relied on self‐reporting, which could produce social desirability bias; participants selecting responses they think would be more acceptable rather than selecting responses based on their true opinions. As this survey was delivered via an online platform there is a chance of selection bias based on participants choosing to participate based on their own interest in the research topic.

## Conclusion

The findings from this study may be considered further validation that education for radiation oncology practitioners in the area of exercise and nutrition as interventions is suitable. Further investigation is required to understand whether this training should be considered as part of their tertiary education. In addition, these results provide an insight into the perceived importance and benefits of a tailored exercise and nutrition programme being implemented for patients with breast cancer receiving RT, as reported by practitioners. Results also demonstrate that if such a programme was available, radiation oncology practitioners may choose to refer patients to support side effects and compromised health‐related quality of life. However, a larger scale survey is required to determine more statistically robust conclusions.

## Conflict of interest

The authors declare no conflict of interest.

## Supporting information


**Data S1.** Supporting Information ‐ survey.

**Table S1a.** Proportion of response options provided by each practitioner group regarding recommending exercise and nutrition as interventions to their patients with breast cancer for radiation side‐effects.
**Table S1b.** Proportion of response options provided by each practitioner group regarding recommending exercise and nutrition as interventions to their patients with breast cancer for comprised health‐related quality of life due to radiation therapy.
**Table S2a.** Practitioners perceptions on their patients benefitting from a tailored exercise and nutrition program.
**Table S2b.** Practitioners perceptions on referring patients to a tailored exercise and nutrition program.
**Table S3a.** Practitioner perceptions on participating in training regarding exercise and nutrition as interventions for patients with radiation therapy side‐effects.
**Table S3b.** Practitioner perceptions on participating in training regarding exercise and nutrition as interventions for patients with compromised health‐related quality of life, during radiation therapy.

## Data Availability

The data that support the findings of this study are available from the corresponding author upon reasonable request.
