# Supplementary material for: Practitioner perceptions on the use of exercise and nutritional interventions for patients with breast cancer receiving radiation therapy
Source: J Med Radiat Sci. 2023 Aug 10;70(4):444–53. doi: 10.1002/jmrs.713 (PMC10715360; doi:10.1002/jmrs.713)
Supplement: Supplementary file 1 — Data S1. Supporting Information ‐ survey. [file JMRS-70-444-s001.pdf]

THE UNIVERSITY OF NEWCASTLE AUSTRALIA

GLOBAL CENTRE FOR RESEARCH AND TRAINING IN RADIATION ONCOLOGY

Practitioner Perceptions on the Use of Exercise and Nutritional Interventions for Patients with Breast Cancer Receiving Radiation Therapy

Thank you for your participation in this survey. The survey will take approx. 10 minutes to complete. You will need to answer all questions and your answers will be saved automatically. Thank you!

* 1. What is your age in years?

- ○ 20 - 24
- ○ 25 - 34
- ○ 35 - 44
- ○ 45 - 54
- ○ Over 55

* 2. What is your gender?

- ○ Male
- ○ Female
- ○ Other

* 3. What is your professional discipline?

- Radiation Oncologist
- Radiation Therapist
- Radiation Oncology Nurse
- Radiation Oncology Registrar

* 4. How many years have you worked in your field?

- 0-5
- 6-10
- 11-15
- 16-20
- 21-30
- More than 30

* 5. In what capacity are you employed?

- Casual
- Part-time
- Full-time

* 6. What is the highest level of education you have completed?

- Doctorate
- Masters
- Honours

- ○ Bachelor
- ○ Associate Diploma
- ○ Certificate
- ○ Fellowship
- ○ Other

If you selected 'other' please specify:

* 7. Please choose which of the following best describes your employment setting (you may choose more than one answer).

- ☐ Metropolitan
- ☐ Rural
- ☐ Public
- ☐ Private
- ☐ Other

If you selected 'other' please specify:

* 8. What state/territory do you work in?

- ○ NSW
- ○ QLD
- ○ NT

- ACT
- VIC
- WA
- TAS
- SA

---

* 9. How many linear accelerators are there in your department?

- 1
- 2
- 3
- 4
- 5
- 6
- 7 or more

---

* 10. How many full time Radiation Therapists work in your department?

- <5
- 6-10
- 11-15
- 16-20
- 21-25
- >25

* 11. What is the standard treatment appointment duration for breast cancer patients?

- ◯ 10 Minutes
- ◯ 12 Minutes
- ◯ 15 Minutes
- ◯ 20 Minutes
- ◯ More than 20 Minutes
- ◯ Unsure
- ◯ Other

If you selected 'other' please specify:

___

* 12. How confident are you regarding your knowledge of ***side-effects*** caused by Radiation Therapy (RT) treatment to the breast?

- ◯ Extremely confident
- ◯ Moderately confident
- ◯ Slightly confident
- ◯ Lacking confidence
- ◯ Not confident

* 13. How confident are you regarding your knowledge of ***Quality of Life*** (QoL) problems caused by RT treatment to the breast?

- ◯ Extremely confident
- ◯ Moderately confident

- ◯ Slightly confident
- ◯ Lacking confidence
- ◯ Not confident

---

14. Do you feel responsible for initiating intervention/s to support patient's side-effects/QoL outcomes?

- ◯ Yes
- ◯ No

---

15. Why do you not feel responsible for initiating intervention/s to support side-effects/QoL outcomes? You may choose more than one answer

- ☐ It is not my role
- ☐ I do not feel confident to do so
- ☐ My workload is too high
- ☐ Not enough time
- ☐ Other

If you selected 'other' please specify:

____

---

16. How often do you assess a patient's ***side-effect/s?*** You may choose more than one answer.

- ☐ Every interaction
- ☐ Every few interactions
- ☐ When I feel it necessary

- [ ] When my colleagues ask me to
- [ ] When the patient asks/mentions it
- [ ] Never

* 17. How do you communicate with the multi-disciplinary team about a patient's ***side-effects?*** You may choose more than one answer.

- [ ] Face to face
- [ ] Email
- [ ] Electronic/paper medical records
- [ ] Phone call
- [ ] Text message
- [ ] Other

If you selected 'other' please specify:

* 18. How often do you ask for a second opinion from your colleagues when assessing a patient's ***side-effects?***

- ○ Always
- ○ Often
- ○ Sometimes
- ○ Never

19. Does the experience level of your colleague change your decision in involving them in your assessment?

- ◯ Always
- ◯ Most of the time
- ◯ About half the time
- ◯ Sometimes
- ◯ Never

* 20. How often do you feel too busy to properly assess a patient's ***side-effects?***

- ◯ Always
- ◯ Often
- ◯ Sometimes
- ◯ Never

* 21. How often do you assess a patient's ***QoL?*** You may choose more than one answer.

- ☐ Every interaction
- ☐ Every few interactions
- ☐ When I feel it necessary
- ☐ When my colleagues ask me to
- ☐ When the patient asks/mentions it
- ☐ Never

* 22. How do you communicate with the multi-disciplinary team about a patient's ***QoL?*** You may choose more than one answer.

- ☐ Face to face
- ☐ Email

- [ ] Electronic/paper medical records
- [ ] Phone call
- [ ] Text message
- [ ] Other

If you selected 'other' please specify:

* 23. How often do you ask for a second opinion from your colleagues when assessing a patient's ***QoL?***

- ◯ Always
- ◯ Often
- ◯ Sometimes
- ◯ Never

* 24. Does the experience level of your colleague change your decision in involving them in your assessment?

- ◯ Always
- ◯ Often
- ◯ Sometimes
- ◯ Never

25. How often do you feel too busy to properly assess a patient's ***QoL?***

- ◯ Always
- ◯ Often

- Sometimes
- Never

---

* 26. How confident are you in your knowledge regarding ***exercise*** as an intervention for RT induced ***side-effects?***

- Extremely confident
- Moderately confident
- Slightly confident
- Lacking confidence
- Not confident

---

* 27. Do you recommend ***exercise*** as a ***side-effect*** intervention to your patients?

- Always
- Most of the time
- About half the time
- Sometimes
- Never

---

* 28. How confident do you feel recommending ***exercise*** as a ***side-effect*** intervention to your patients?

- Extremely confident
- Moderately confident
- Slightly confident
- Lacking confidence

- ◯ Not confident

* 29. When do you recommend ***exercise*** as a ***side-effect*** intervention to your patients?

- ◯ Always
- ◯ Once they have mentioned a problem that would benefit from it
- ◯ When I think they will benefit from it
- ◯ Never
- ◯ Other

If selected 'other' please specify:

* 30. Do you feel you would benefit from more training in the area of ***exercise*** as a ***side-effect*** intervention?

- ◯ Definitely yes
- ◯ Probably yes
- ◯ Probably not
- ◯ Definitely not
- ◯ Unsure

* 31. If it was offered to you, would you take part in more training in the area of ***exercise*** as a ***side-effect*** intervention?

- ◯ Definitely yes
- ◯ Probably yes

- Probably not
- Definitely not
- Unsure

---

* 32. How confident are you in your knowledge regarding ***nutrition/diet*** as an intervention for RT induced ***side-effects?***

- Extremely confident
- Moderately confident
- Slightly confident
- Lacking confidence
- Not confident

---

* 33. Do you recommend your patients seek ***nutrition/dietary advice*** as a ***side-effect*** intervention?

- Always
- Most of the time
- About half the time
- Sometimes
- Never

---

* 34. How confident do you feel recommending ***nutrition/dietary advice*** as a ***side-effect*** intervention to your patients?

- Extremely confident
- Moderately confident
- Slightly confident

- Lacking confidence
- Not confident

* 35. When do you recommend ***nutrition/dietary advice*** as a ***side-effect*** intervention to your patients?

- Always
- Once they have mentioned a problem that would benefit from it
- When I think they will benefit from it
- Never
- Other

If you selected 'other' please specify:

* 36. Do you feel you would benefit from more training in the area of ***nutrition/diet*** as a ***side-effect*** intervention?

- Definitely yes
- Probably yes
- Probably not
- Definitely not
- Unsure

* 37. If it was offered to you, would you take part in more training in the area of ***nutrition/diet*** as a ***side-effect*** intervention?

- Definitely yes

- Probably yes
- Probably not
- Definitely not
- Unsure

* 38. How confident are you in your knowledge regarding ***exercise*** as an intervention for RT induced ***QoL*** problems?

- Extremely confident
- Moderately confident
- Slightly confident
- Lacking confidence
- Not confident

* 39. Do you recommend ***exercise*** as a ***QoL*** intervention to your patients?

- Always
- Most of the time
- About half the time
- Sometimes
- Never

* 40. How confident do you feel recommending ***exercise*** as a ***QoL*** intervention to your patients?

- Extremely confident
- Moderately confident

◯ Slightly confident

◯ Lacking confidence

◯ Not confident

---

* 41. When do you recommend ***exercise*** as a ***QoL*** intervention to your patients?

◯ Always

◯ Once they have mentioned a problem that would benefit from it

◯ When I think they would benefit from it

◯ Never

◯ Other

If selected 'other' please specify:

____

---

* 42. Do you feel you would benefit from more training in the area of ***exercise*** as a ***QoL*** intervention?

◯ Definitely yes

◯ Probably yes

◯ Probably not

◯ Definitely not

◯ Unsure

---

* 43. If it was offered to you, would you take part in more training in the area of ***exercise*** as a ***QoL*** intervention?

◯ Definitely yes

- Probably yes
- Probably not
- Definitely not
- Unsure

---

* 44. How confident are you in your knowledge regarding **diet** as an intervention for RT induced **QoL problems?**

- Extremely confident
- Moderately confident
- Slightly confident
- Lacking confidence
- Not confident

---

* 45. Do you recommend ***nutrition/dietary advice*** as a ***QoL*** intervention to your patients?

- Always
- Most of the time
- About half the time
- Sometimes
- Never

---

* 46. How confident do you feel recommending ***nutrition/dietary advice*** as a ***QoL*** intervention to your patients?

- Extremely confident
- Moderately confident

- Slightly confident
- Lacking confidence
- Not confident

* 47. When do you recommend ***nutrition/dietary advice*** as a ***QoL*** intervention to your patients?

- Always
- Once they have mentioned a problem that would benefit from it
- When I think they would benefit from it
- Never
- Other

If you selected 'other' please specify:

* 48. Do you feel you would benefit from more training in the area of ***nutrition/diet*** as a ***QoL*** intervention?

- Definitely yes
- Probably yes
- Probably not
- Definitely not
- Unsure

* 49. If it was offered to you, would you take part in more training in the area of ***nutrition/diet*** as a ***QoL*** intervention?

- Definitely yes
- Probably yes
- Probably not
- Definitely not
- Unsure

* 50. Do you think your patients would benefit from a customised exercise and nutrition program?

- Definitely yes
- Probably yes
- Probably not
- Definitely not
- Unsure

* 51. If available, would you refer your patients to a customised exercise and nutrition program?

- Definitely yes
- Probably yes
- Might or might not
- Probably not
- Definitely not
