# Supplementary material for: Practitioner perceptions on the use of exercise and nutritional interventions for patients with breast cancer receiving radiation therapy
Source: J Med Radiat Sci. 2023 Aug 10;70(4):444–53. doi: 10.1002/jmrs.713 (PMC10715360; doi:10.1002/jmrs.713)
Supplement: Supplementary file 2 — Table S1a. Proportion of response options provided by each practitioner group regarding recommending exercise and nutrition as interventions to their patients with breast cancer for radiation side‐effects. Table S1b. Proportion of response options provided by each practitioner group regarding recommending exercise and nutrition as interventions to their patients with breast cancer for comprised health‐related quality of life due to radiation therapy. Table S2a. Practitioners perceptions on their patients benefitting from a tailored exercise and nutrition program. Table S2b. Practitioners perceptions on referring patients to a tailored exercise and nutrition program. Table S3a. Practitioner perceptions on participating in training regarding exercise and nutrition as interventions for patients with radiation therapy side‐effects. Table S3b. Practitioner perceptions on participating in training regarding exercise and nutrition as interventions for patients with compromised health‐related quality of life, during radiation therapy. [file JMRS-70-444-s002.docx]

**Supporting Information Table 1a.** Proportion of response options provided by each practitioner group regarding recommending exercise and nutrition as interventions to their patients with breast cancer for radiation side-effects.

|  | ROs (E)  N (%) | ROs (N)  N (%) | RORs (E)  N (%) | RORs (N)  N (%) | RTTs (E)  N (%) | RTTs (N)  N (%) | RONs (E)  N (%) | RONs (N)  N (%) |
| --- | --- | --- | --- | --- | --- | --- | --- | --- |
| Always | 3 (27%) | 2 (18%) | 0 (0%) | 0 (0%) | 0 (0%) | 5 (13%) | 6 (27%) | 3 (14%) |
| Most of the time | 5 (45%) | 4 (36%) | 1 (33%) | 0 (0%) | 4 (10%) | 11 (28%) | 8 (36%) | 12 (55%) |
| About half of the time | 0 (0%) | 1 (9%) | 1 (33%) | 0 (0%) | 2 (5%) | 2 (5%) | 3 (14%) | 1 (5%) |
| Sometimes | 3 (27%) | 3 (27%) | 1 (33%) | 3 (100%) | 29 (73%) | 18 (45%) | 3 (14%) | 4 (18%) |
| Never | 0 (0%) | 1 (9%) | 0 (0%) | 0 (0%) | 5 (13%) | 4 (10%) | 2 (9%) | 2 (9%) |

**Supporting Information Table 1b.** Proportion of response options provided by each practitioner group regarding recommending exercise and nutrition as interventions to their patients with breast cancer for comprised health-related quality of life due to radiation therapy.

|  | ROs (E)  N (%) | ROs (N)  N (%) | RORs (E)  N (%) | RORs (N)  N (%) | RTTs (E)  N (%) | RTTs (N)  N (%) | RONs (E)  N (%) | RONs (N)  N (%) |
| --- | --- | --- | --- | --- | --- | --- | --- | --- |
| Always | 4 (36%) | 3 (27%) | 1 (33%) | 0 (0%) | 1 (3%) | 0 (0%) | 6 (27%) | 5 (23%) |
| Most of the time | 2 (18%) | 3 (27%) | 1 (33%) | 1 (33%) | 4 (10%) | 3 (8%) | 9 (41%) | 11 (50%) |
| About half of the time | 2 (18%) | 0 (0%) | 1 (33%) | 0 (0%) | 5 (13%) | 5 (13%) | 4 (18%) | 2 (9%) |
| Sometimes | 3 (27%) | 4 (36%) | 0 (0%) | 2 (67%) | 26 (65%) | 21 (53%) | 3 (14%) | 4 (18%) |
| Never | 0 (0%) | 1 (9%) | 0 (0%) | 0 (0%) | 4 (10%) | 11 (28%) | 0 (0%) | 0 (0%) |

**Supporting Information Table 2a.** Practitioners perceptions on their patients benefitting from a tailored exercise and nutrition program.

|  | ROs  N (%) | RORs  N (%) | RTTs  N (%) | RONs  N (%) |
| --- | --- | --- | --- | --- |
| Definitely yes | 4 (36%) | 0 (0%) | 23 (58%) | 9 (41%) |
| Probably yes | 7 (64%) | 3 (100%) | 16 (40%) | 12 (55%) |
| Probably not | 0 (0%) | 0 (0%) | 0 (0%) | 1 (5%) |
| Definitely not | 0 (0%) | 0 (0%) | 0 (0%) | 0 (0%) |
| Unsure | 0 (0%) | 0 (0%) | 1 (3%) | 0 (0%) |

**Supporting Information Table 2b.** Practitioners perceptions on referring patients to a tailored exercise and nutrition program.

|  | ROs  N (%) | RORs  N (%) | RTTs  N (%) | RONs  N (%) |
| --- | --- | --- | --- | --- |
| Definitely yes | 5 (45%) | 0 (0%) | 27 (68%) | 15 (68%) |
| Probably yes | 5 (45%) | 2 (67%) | 11 (28%) | 5 (23%) |
| Probably not | 1 (9%) | 1 (33%) | 2 (5%) | 2 (9%) |
| Definitely not | 0 (0%) | 0 (0%) | 0 (0%) | 0 (0%) |
| Unsure | 0 (0%) | 0 (0%) | 0 (0%) | 0 (0%) |

**Supporting Information Table 3a.** Practitioner perceptions on participating in training regarding exercise and nutrition as interventions for patients with radiation therapy side-effects.

|  | ROs (E)  N (%) | ROs (N)  N (%) | RORs (E)  N (%) | RORs (N)  N (%) | RTTs (E)  N (%) | RTTs (N)  N (%) | RONs (E)  N (%) | RONs (N)  N (%) |
| --- | --- | --- | --- | --- | --- | --- | --- | --- |
| Definitely yes | 2 (18%) | 2 (18%) | 2 (67%) | 3 (100%) | 29 (73%) | 28 (70%) | 14 (64%) | 12 (55%) |
| Probably yes | 8 (73%) | 7 (64%) | 1 (33%) | 0 (0%) | 9 (23%) | 11 (28%) | 8 (36%) | 10 (45%) |
| Probably not | 1 (9%) | 1 (9%) | 0 (0%) | 0 (0%) | 1 (3%) | 0 (0%) | 0 (0%) | 0 (0%) |
| Definitely not | 0 (0%) | 1 (9%) | 0 (0%) | 0 (0%) | 0 (0%) | 0 (0%) | 0 (0%) | 0 (0%) |
| Unsure | 0 (0%) | 0 (0%) | 0 (0%) | 0 (0%) | 1 (3%) | 1 (3%) | 0 (0%) | 0 (0%) |

**Supporting Information Table 3b.** Practitioner perceptions on participating in training regarding exercise and nutrition as interventions for patients with compromised health-related quality of life, during radiation therapy.

|  | ROs (E)  N (%) | ROs (N)  N (%) | RORs (E)  N (%) | RORs (N)  N (%) | RTTs (E)  N (%) | RTTs (N)  N (%) | RONs (E)  N (%) | RONs (N)  N (%) |
| --- | --- | --- | --- | --- | --- | --- | --- | --- |
| Definitely yes | 2 (18%) | 2 (18%) | 2 (67%) | 2 (67%) | 27 (68%) | 27 (68%) | 14 (64%) | 12 (55%) |
| Probably yes | 8 (73%) | 7 (64%) | 1 (33%) | 1 (33%) | 11 (28%) | 12 (30%) | 8 (36%) | 9 (41%) |
| Probably not | 1 (9%) | 2 (18%) | 0 (0%) | 0 (0%) | 0 (0%) | 0 (0%) | 0 (0%) | 1 (5%) |
| Definitely not | 0 (0%) | 0 (0%) | 0 (0%) | 0 (0%) | 0 (0%) | 0 (0%) | 0 (0%) | 0 (0%) |
| Unsure | 0 (0%) | 0 (0%) | 0 (0%) | 0 (0%) | 2 (5%) | 1 (3%) | 0 (0%) | 0 (0%) |

**Abbreviations:**

- Radiation Oncologists = ROs
- Radiation Oncology Registrars = RORs
- Radiation Therapists = RTTs
- Radiation Oncology Nurses = RONs
- Exercise = (E)
- Nutrition = (N)
